# Techno-economic data and assumptions for long-term energy systems modelling in Viet Nam

**DOI:** 10.1016/j.dib.2022.108836

**Published:** 2022-12-17

**Authors:** Naomi Tan, John Harrison, Mark Howells, Rudolf Yeganyan

**Affiliations:** a Centre for Sustainable Transitions: Energy, Environment and Resilience, Loughborough University, United Kingdom; b Centre for Environmental Policy, Imperial College London, United Kingdom

**Keywords:** Renewable energy, Cost-optimization, Energy policy, OSeMOSYS

## Abstract

Viet Nam is at a critical juncture in planning for its future energy mix due to its fast-growing economy and recent climate commitments. Robust modelling analyses examining the potential and practical energy mix alternatives are therefore key in providing key stakeholders with critical information on energy policy decision-making. The challenge is that a large volume of data is required to accurately model various energy pathways at the national scale. This data note, therefore, aims to bridge the current data gap by providing key information on input data and assumptions for long-term energy planning in Viet Nam. Historical and/or projected data regarding electricity generation and consumption, electricity imports and exports, fuel prices, emissions, refineries, power transmission and distribution, electricity generation technologies, and renewable energy potential and reserves for the years 2015 to 2050 are described in this article.

**Specifications Table**

**Table:** 

Subject	Energy
Specific subject area	Energy System Modelling
Type of data	Table Graph
How the data were acquired	Literature survey (databases and reports from international organisations; journal articles)
Data format	Raw Analyzed
Description of data collection	Data were collected from the websites, annual reports, and databases of international organisations, as well as from academic articles and existing modelling databases. Data were collected and manipulated based on the inputs required to build an energy system model on the linear cost-optimization tool called OSeMOSYS. Nonetheless, the data available through this document is independent of the tool.
Data source location	Raw data sources are listed in Table 1 of this article.
Data accessibility	With this article and in a repository. Repository name: Zenodo Data identification number: 10.5281/zenodo.7276392 Direct URL to data: https://doi.org/10.5281/zenodo.7276392.

**Value of the Data**

• These data can be used to develop energy system models in Viet Nam to inform national energy investment outlooks and policy plans. Insights into the evolution of the electricity supply system under different trajectories can also be provided.
• The data are useful for country analysts, policymakers, and the broader scientific community, as a base for model development.
• These data could be used to examine a range of possible energy system pathways to provide further insights into the evolution of Viet Nam's power system.
• The data are open-source and country-specific which is not easily accessible in current literature.
• The data can be used both for conducting analyses of Viet Nam's power system and for capacity-building activities.
• By combining secondary data from multiple, diverse sources, the work provides analysts with complete and accessible datasets, helping to overcome barriers of data inaccessibility.

## 1 Objective

Energy modelling analyses examining the potential and practical energy pathways for decarbonization planning are important in providing key stakeholders with critical information on energy policy decision-making. The challenge is that a large volume of data is required to accurately model various energy pathways at the national scale. However, data may be inaccessible, outdated, inconsistent, and lacking quality. Furthermore, the collection of such may be tiresome and time-consuming.

As a result, this article aims to bridge the current data gap by providing key information on input data and assumptions for long-term energy planning in Viet Nam. The data can be used by academics, consultants, or government officials for further energy systems modelling in Viet Nam or Asia. The dataset promotes the U4RIA goals [1], which are Ubuntu, Retrievability, Reusability, Repeatability, Reconstructability, Interoperability, and Auditability. The goals note how “energy modelling that provides policy support should not only be grounded in rigorous analytics, but also in good governance principles”. Further, it should be accountable with other policy actions. Overall, these goals and data are designed to improve energy modelling for policy support.

## 2 Data Description

This paper presents selected country-specific data which can be used in the Open Source Energy Modelling System (OSeMOSYS) tool for long-term energy decarbonization planning. Nonetheless, the data available through this document is independent of the tool. The data provided were collected from publicly available sources, including the reports of international organisations, journal articles and existing model databases (Table 1). It includes historical and/or projected data regarding electricity generation and consumption, electricity imports and exports, fuel prices, emissions, refineries, power transmission and distribution, electricity generation technologies, and renewable energy potential and reserves from 2015 to 2050.

**Table 1:** List of sources used in this article.

Source	Reference
APEC Energy Demand and Supply Outlook 8th Edition 2022	APEC, “APEC Energy Demand and Supply Outlook 8th Edition,” Tokyo, 2022. [Online]. Available: https://aperc.or.jp/reports/outlook.php
BP Statistical Review of World Energy 2022	BP, “Statistical Review of World Energy,” London, 2022. [Online]. Available: https://www.bp.com/en/global/corporate/energy-economics/statistical-review-of-world-energy/downloads.html
Vietnam Energy Outlook Report 2019	EREA & DEA, “Vietnam Energy Outlook Report,” Hanoi, 2019. [Online]. Available: https://ens.dk/sites/ens.dk/files/Globalcooperation/vietnam_energy_outlook_report_2019.pdf
Vietnam Energy Outlook Report 2022	EREA & DEA, “Vietnam Energy Outlook Report,” Hanoi, 2022. [Online]. Available: https://ens.dk/sites/ens.dk/files/Globalcooperation/vietnam_energy_outlook_report_2021_english.pdf
IEA Country Profile	IEA, “Viet Nam,” International Energy Agency, 2019. https://www.iea.org/countries/viet-nam (accessed Nov. 02, 2022).
IEA Country Balance	IEA, “Viet Nam Balance,” International Energy Agency, 2020. https://www.iea.org/sankey/#?c=Viet Nam&s=Balance (accessed Nov. 02, 2022).
IRENA Renewable Power Generation Costs in 2021	IRENA, “Renewable Power Generation Costs in 2021,” Abu Dhabi, 2022. https://www.irena.org/-/media/Files/IRENA/Agency/Publication/2022/Jul/IRENA_Power_Generation_Costs_2021.pdf?rev=34c22a4b244d434da0accde7de7c73d8
IRENASTAT Power Capacity and Generation	IRENA, “IRENASTAT Power Capacity and Generation,” International Renewable Energy Agency, 2022. https://www.irena.org/Data/Downloads/IRENASTAT (accessed Nov. 02, 2022).

### 2.1 Electricity demand

Historical data on Viet Nam's annual electricity demand from the year 2015 to 2018 and projected data from 2019 to 2050 is from the APEC Energy Demand and Supply Outlook 8th Edition [2]. The latter is based on the report's Reference scenario. Table 2 notes down the electricity demand for key years. The electricity demand assumes no influence from unforeseen circumstances such as pandemics or natural disasters.

**Table 2:** Electricity demand for key years in PJ.

2015	2020	2025	2030	2035	2040	2045	2050
593.956	852.445	1140.179	1430.025	1767.842	2182.377	2614.525	3100.973

**Fig. 1:**
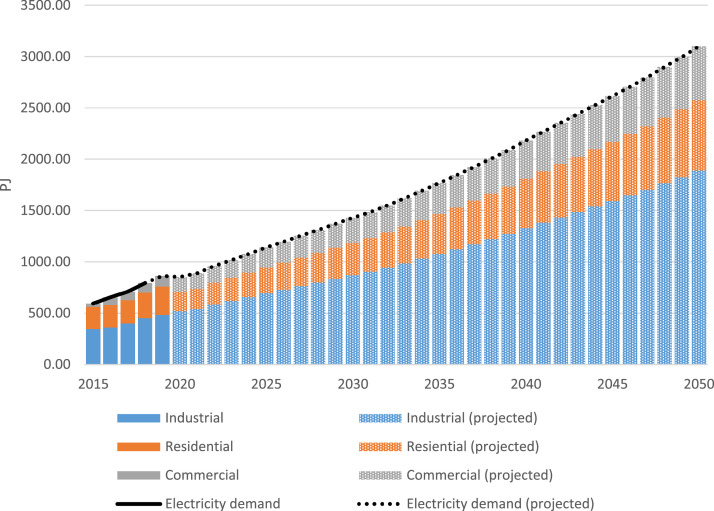
Electricity demand by sector.

Electricity demand can be broken down into sectors such as industrial, residential, and commercial. Historical data for these sectors were gathered from the IEA Country Profiles [3] and projected with data from the 2022 Vietnam Energy Outlook Report [4]. The latter noted that the energy service demands are expected to increase at the same rate in the residential, commercial, and industrial sectors from 2020 to 2050. Thus, the sectors are shown in Table 3 and Fig. 1 with the same increase rate after 2020.

**Table 3:** Electricity demand by sector for key years in PJ.

	2015	2020	2025	2030	2035	2040	2045	2050
Industrial	345.47	519.42	694.31	870.81	1076.52	1328.95	1592.11	1888.33
Residential	216.11	188.31	251.72	315.71	390.29	481.80	577.21	684.60
Commercial	32.380	145.25	94.15	243.51	301.03	371.62	445.21	528.04

### 2.2 Electricity generation by source

**Fig. 2:**
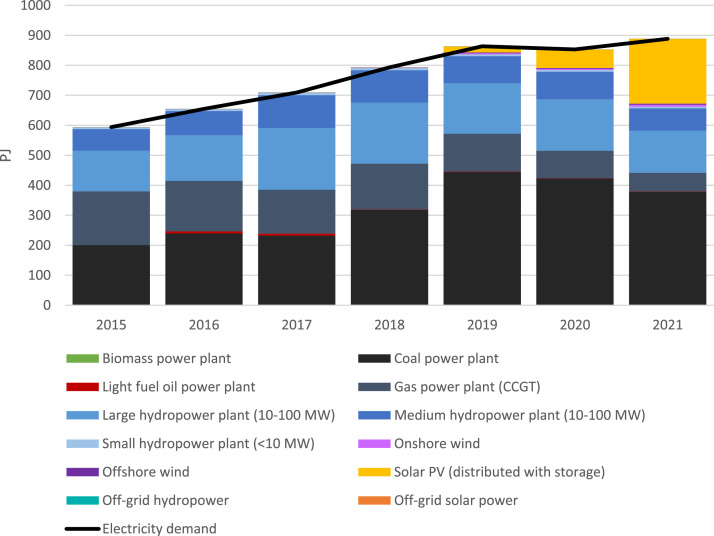
Annual electricity generation by source and electricity demand.

The annual electricity generation from 2015 to 2021 is listed in Table 4. Data were collected from the IRENASTAT database [5] and processed to show the generation by sub-technologies. This is plotted against the total electricity generation in Fig. 2.

**Table 4:** Annual electricity generation (2015-2021) by source in PJ.

Sub-technology	2015	2016	2017	2018	2019	2020	2021
Biomass power plant	1.52	1.37	1.58	1.70	1.61	1.18	1.07
Coal power plant	199.33	238.21	230.76	316.30	442.63	421.24	378.00
Light fuel oil power plant	0.00	6.75	6.66	0.93	1.05	1.00	0.85
Gas power plant (CCGT)	179.39	168.35	146.18	153.47	126.69	92.02	62.05
Large hydropower plant (10-100 MW)	135.54	152.32	205.81	203.19	168.88	171.71	140.25
Medium hydropower plant (10-100 MW)	72.09	81.01	109.46	108.07	89.82	91.32	74.59
Small hydropower plant (<10 MW)	4.99	5.60	7.57	7.47	6.21	6.32	5.16
Onshore wind	0.18	0.30	0.51	0.68	3.56	3.78	6.45
Offshore wind	0.48	0.49	0.48	0.49	2.66	2.83	4.02
Solar PV (distributed with storage)	0.00	0.00	0.01	0.38	19.86	61.14	215.72
Off-grid hydropower	0.42	0.42	0.42	0.43	0.45	0.41	0.32
Off-grid solar power	0.02	0.02	0.02	0.02	0.02	0.02	0.01

### 2.3 Electricity imports and exports

**Fig. 3:**
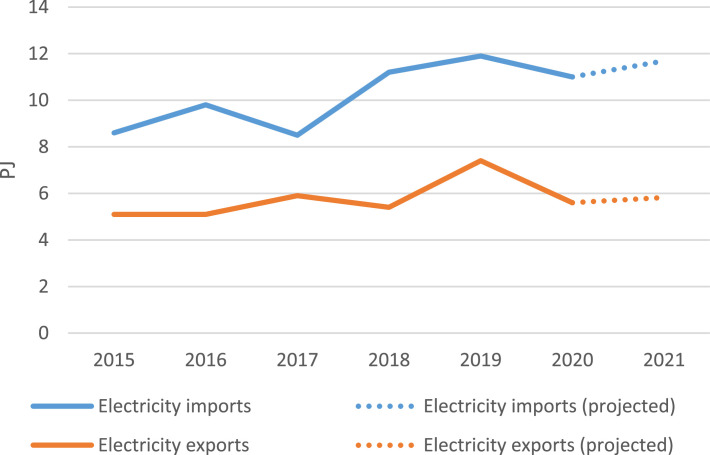
Annual imports and exports (2015-2021).

Historical data on electricity imports and exports from 2015 to 2020 were taken from the IEA Country Balance [6] and noted in Table 5. Due to unavailable open data for the year 2021, this value was projected based on the average annual growth rate from 2015 onwards. The upward trend of electricity imports and exports can be seen in Fig. 3.

**Table 5:** Annual electricity imports and exports (2015-2021) in PJ.

	2015	2016	2017	2018	2019	2020	2021
Imports	8.6	9.8	8.5	11.2	11.9	11	11.7
Exports	5.1	5.1	5.9	5.4	7.4	5.6	5.8

**Fig. 4:**
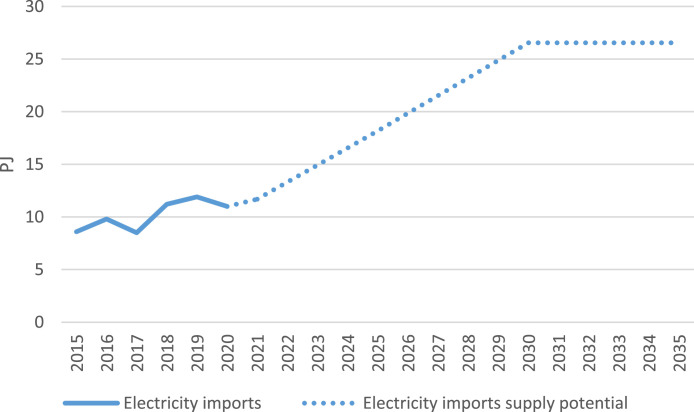
Electricity imports supply potential.

Further, the 2019 Vietnam Energy Outlook Report [7] noted that electricity imports in Viet Nam have the potential increase rapidly, reaching a maximum supply potential of 26.55 PJ by 2030. The Energy Outlook Report also states that electricity imports in the country will stay at this level after 2030 (Fig. 4).

**Fig. 5:**
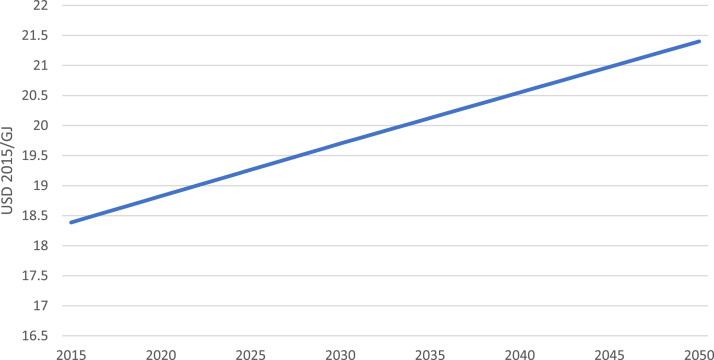
Electricity import price.

Additionally, projected electricity import prices were taken from the 2019 Vietnam Energy Outlook Report [7]. The average electricity import price of the country was taken to show the overall price increase from 2015 to 2050. Further, the electricity import price prior to 2020 was calculated based on the average annual increase rate, due to lack of available open data (Table 6, Fig. 5).

**Table 6:** Electricity import price for key years in USD 2015/GJ.

2015	2020	2025	2030	2035	2040	2045	2050
18.388	18.825	19.263	19.700	20.125	20.550	20.975	21.400

### 2.4 Fuel prices

**Fig. 6:**
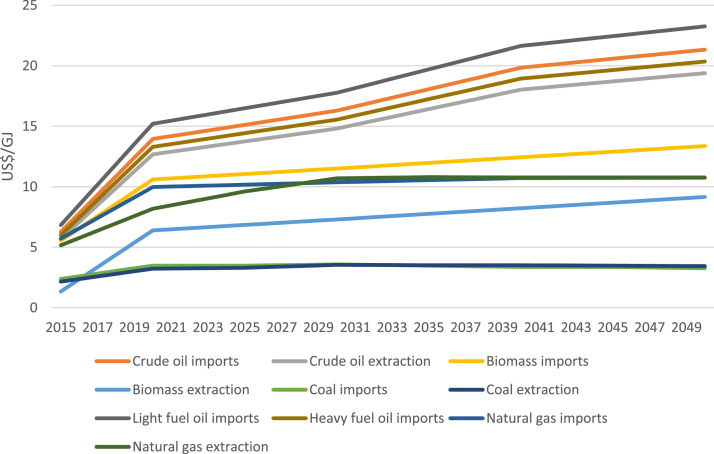
Fuel prices.

Data regarding the price of oil imports and extraction were gathered from the Climate Compatible Growth (CCG) Starter Kit [8], while coal and natural gas imports and extractions were from an appendix report of the 2022 Vietnam Energy Outlook Report [9], and biomass import and extraction were based on data from the 2019 Vietnam Energy Outlook Report [7]. However, 2015 values for biomass, coal, and gas were taken from the CCG Starter Kit. The methodology applied to the biomass, coal, and gas calculation is noted in more detail in Section 3.4. Table 7 and Fig. 6 displays the fuel price for key dates and projection, respectively.

**Table 7:** Fuel prices for key years in USD/GJ.

	2015	2020	2025	2030	2035	2040	2045	2050
Crude oil imports	6.27	13.95	15.12	16.29	18.07	19.84	20.59	21.33
Crude oil extraction	5.7	12.68	13.75	14.81	16.42	18.03	18.71	19.39
Biomass imports	5.55	10.60	11.06	11.51	11.97	12.44	12.90	13.36
Biomass extraction	1.34	6.39	6.85	7.30	7.76	8.23	8.69	9.15
Coal imports	2.38	3.48	3.48	3.58	3.48	3.37	3.37	3.26
Coal extraction	2.16	3.23	3.3	3.55	3.51	3.51	3.48	3.44
Light fuel oil imports	6.83	15.21	16.49	17.77	19.71	21.64	22.45	23.26
Heavy fuel oil imports	5.99	13.3	14.43	15.55	17.25	18.94	19.65	20.35
Natural gas imports	5.71	9.98	10.17	10.37	10.55	10.72	10.74	10.75
Natural gas extraction	5.16	8.18	9.61	10.70	10.80	10.76	10.76	10.76

### 2.5 Emissions

Power plants emit greenhouse gases over their lifetime, such as carbon dioxide, methane, and nitrous oxides. In this study, the emissions are noted as carbon dioxide equivalent (CO_2_e), displayed in Table 8. Data for crude, light fuel, and heavy fuel oil were taken from an ICCT report [10], whilst data for biomass, coal, and natural gas were taken from an IPCC annex [11]. Median values were taken from both documents.

**Table 8:** Emission factor of CO_2_e for various fuel types.

Fuel	Emission factor (kg CO2e/GJ)
Crude oil	27
Biomass	134.7
Coal	227.8
Light Fuel Oil	100
Heavy Fuel Oil	100
Natural Gas	136.1

### 2.6 Refineries

Data on refineries were gathered from a European Union report on energy projections [12]. Although these values are for an African energy system, it is assumed to be representative of Viet Nam due to lack of available data. Further, they are assumed to stay constant. This is noted in Table 9.

**Table 9:** Techno-economic data on refineries.

Technology	Capital cost (USD/kW) in 2019	Variable cost (USD/GJ) in 2019	Operational life (years)	Output ratio
Crude oil refinery option 1	24.1	0.71775	35	0.9 LFO: 0.1 HFO
Crude oil refinery option 2	24.1	0.71775	35	0.8 LFO: 0.2 HFO

### 2.7 Power transmission and distribution

Data on the power transmission and distribution system is also needed for the modelling and analysis of energy systems. Data on costs and operational life were collected by calculating the average value of nine power transmission lines in the Greater Mekong Subregion [13], and is assumed to stay constant due to lack of available open data. Data on efficiency of the power transmission and distribution system was based on Index Mundi [14]. Table 10 and Table 11 display the data.

**Table 10:** Techno-economic data on the power transmission and distribution system.

Capital cost (USD/kW) in 2014	Fixed cost (USD/kW/yr) in 2014	Operational life (years)
306.43	6.13	40

**Table 11:** Efficiency of the power transmission and distribution system for key years.

2015	2020	2025	2030	2035	2040	2045	2050
0.908	0.914	0.920	0.926	0.932	0.938	0.944	0.950

### 2.8 Electricity generation technologies

**Fig. 7:**
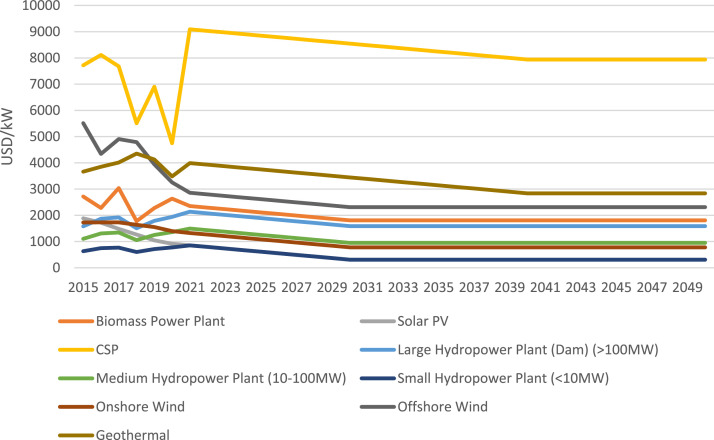
Capital cost of renewable electricity generation technologies.

Data regarding electricity generation technologies are collected from the CCG Starter Kit [8] and are shown in Table 12. It is assumed that the performance of the technologies in Table 12 stays constant due to lack of available data. Global historical capital costs of renewable electricity generation technologies from 2015 to 2021 were also extracted from IRENA's report on Renewable Power Generation Costs [15] and projected to 2050. This is noted in Table 13 and Fig. 7.

**Table 12:** Techno-economic data on the electricity generation technologies.

Technology	Capital cost (USD/kW) in 2020	Fixed cost (USD/kW/yr) in 2020	Operational life (years)	Efficiency	Average capacity factor
Biomass Power Plant	2750	69	25	0.38	0.7
Coal Power Plant	1300	52	60	0.3	0.75
Geothermal Power Plant	2500	100	50	0.1	0.7
Light Fuel Oil Power Plant	1200	18	50	0.4	0.25
Oil Fired Gas Turbine (SCGT)	1344	18	50	0.4	0.25
Gas Power Plant (CCGT)	1000	40	30	0.55	0.55
Gas Power Plant (SCGT)	784	23	30	0.35	0.55
Solar PV (Utility)	1160	15.08	30	1	0.23
CSP with Storage	4965.31	120	35	0.33	0.3
Large Hydropower Plant (Dam) (>100MW)	1539	46.17	40	1	0.49
Medium Hydropower Plant (10-100MW)	1592.86	47.79	40	1	0.49
Small Hydropower Plant (<10MW)	2162	64.86	40	1	0.49
Onshore Wind	2220.09	88.8	30	1	0.15
Offshore Wind	2876.21	115.05	30	1	0.27
Nuclear Power Plant	5500	138	60	0.33	0.83
Light Fuel Oil Standalone Generator (1kW)	1500	38	20	0.42	0.4
Solar PV (Distributed with Storage)	2130.8	42.62	24	1	0.23

**Table 13:** Capital cost of renewable electricity generation technologies for key years in 2021 USD/kW.

Technology	2015	2020	2025	2030	2035	2040	2045	2050
Biomass Power Plant	2717	2634	2110	1807	1807	1807	1807	1807
Solar PV	1887	916	614	311	311	311	311	311
CSP	7718	4746	8848	8545	8242	7938	7938	7938
Large Hydropower Plant (Dam) (>100MW)	1579	1939	1892	1589	1589	1589	1589	1589
Medium Hydropower Plant (10-100MW)	1105.3	1357.3	1252	949	949	949	949	949
Small Hydropower Plant (<10MW)	631.6	775.6	611	308	308	308	308	308
Onshore Wind	1725	1397	1082	779	779	779	779	779
Offshore Wind	5515	3255	2615	2312	2312	2312	2312	2312
Geothermal	3665	3483	3748	3445	3142	2838	2838	2838

### 2.9 Renewable energy supply potential and reserves

In this study, data on domestic renewable energy supply potential and fossil fuel reserves are taken from available open reports and databases. In more detail, biomass supply potential is an average taken from the 2019 Vietnam Energy Outlook Report [7]. Geothermal, solar, and wind potentials are from the CCG Starter Kit [8]. On-grid hydro and off-grid hydropower are from EVN [16] and IRENASTAT [5], respectively. Lastly, data for fossil fuel reserves are from the BP Statistical Review of World Energy [17]. It is assumed that all existing and future power plants are allowed to contribute to the reserves. These reserves do not include any contribution from associated reserves in neighbouring countries. Table 14 and Table 15 list the estimated renewable energy potentials and fossil fuel reserves, respectively.

**Table 14:** Renewable energy supply potential for key years.

Fuel	Unit	2020	2030	2050
Biomass	PJ	40.44333	45.18966	74.08
Geothermal	GW	0.4	0.4	0.4
Hydro (large)	GW	24.22519	24.22519	24.22519
Hydro (med)	GW	12.88379	12.88379	12.88379
Hydro (small)	GW	0.891024	0.891024	0.891024
Hydro (off-grid)	GW	0.043	0.043	0.043
Solar PV	PJ	13388.04	13388.04	13388.04
Wind (onshore)	PJ	2289.96	2289.96	2289.96
Wind (offshore)	PJ	16394.15	16394.15	16394.15

**Table 15:** Fossil fuel reserves.

Fuel	Unit	Proven reserves
Coal	million tonnes	3360
Crude oil	billion barrels	4.4
Natural gas	trillion cubic metres	0.6

## 3 Experimental Design, Materials and Methods

Data was collected through an extensive literature review. This included material from international organizations, journal articles, databases, and media reports. The data was compiled, processed, presented, and discussed internally to reach consensus on the main data and assumptions to be used in the analysis. The data sources and method of processing for certain techno-economic parameters are detailed in this section.

### 3.1 Electricity demand

The final electricity demand projection and its historical trend were taken from the Reference scenario of the APEC Energy Demand and Supply Outlook 8^th^ Edition [2]. From this source, the total demand for every five years from 2015 to 2050 were given. Thus, it is assumed that demand will change linearly between these data points.

Electricity consumption by sector for the years 2015 to 2019 were collected from the IEA Country Profiles [3]. The 2020 values were calculated based on the average annual growth rate from 2015 to 2019. This therefore assumes the 2020 value would increase based on previous growth rates. Additionally, the proportion in percentage of electricity consumed by each sector was calculated for the year 2020. As the Vietnam Energy Outlook Report [4] estimated that the growth of the three sectors will be roughly the same from 2020 until 2050, the proportions of the sectors from 2020 are used for the years thereafter.

### 3.2 Electricity generation by source

The electricity generation by source for the years 2015 to 2020 was first compiled from IRENASTAT [5]. As electricity generation data for 2021 was not provided from the database, the electricity generation proportion of all generation technologies, except on-grid solar photovoltaic (PV), were calculated based on the average annual growth rates from 2015 to 2020. Thus, this assumes that electricity generation by source will change based on previous growth trends. As the 2019 to 2020 growth trend of on-grid solar PV from IRENASTAT [5] increased far more than previous years, the 2020 to 2021 growth trend of solar PV from Ember [18] was used instead, to avoid an anomaly calculation when incorporating the average annual growth rate. The data for electricity generation by on-grid solar PV therefore combines two sources. Nonetheless, the value is in the same order of magnitude and considered acceptable by the authors. The proportion in percentage of annual electricity generated by each sub-technology was calculated and processed to align with the overall electricity generation data from the APEC Energy Demand and Supply Outlook 8^th^ Edition [2]. Lastly, on-grid hydropower generation was split into different sizes based on the existing capacity of large, medium, and small sized hydropower plants [19].

### 3.3 Electricity imports

The electricity supply potential was compiled and averaged from the Vietnam Energy Outlook Report, which gave data for the years 2020, 2030, and 2050 [7]. From this, data for the year 2025 was calculated as the mid-point value from 2020 to 2030. As the average estimated supply potential for the year 2020 was lower than what was recorded by the IEA [6], the initial value is discarded and the latter incorporated instead. The average data for 2025 still remains as an estimate projection nonetheless. From the averages, it is assumed that demand will change linearly between these data points.

The electricity import price was compiled and averaged from the Vietnam Energy Outlook Report [7]. From the averages, it is assumed that demand will change linearly between these data points. Further, due to lack of available open data before the year 2020, the price of imported electricity before 2020 is assumed to have the same linear trend as mentioned above.

### 3.4 Fuel prices

Biomass extraction prices by region and by fuel type were collected and averaged from the Vietnam Energy Outlook [7]. From the averages, it is assumed that prices will change linearly between these data points. This is the same for coal and gas imports and extraction [9] and oil imports and exports [8]. As the Vietnam Energy Outlook Reports [4, 7] does not provide data for the years before 2020, the price of biomass,coal, and gas in 2015 is taken from the CCG Starter Kit [8], and linearly scaled to their respective 2020 values. Further, the price of imported biomass is expected to increase with the same growth rate as extracted biomass due to lack of available data.

### 3.5 Power transmission and distribution

The percentage loss of output in the power transmission and distribution system for the year 2014 was collected from Index Muni [14] and used to calculate the efficiency of the system. It was then assumed the percentage of loss will reduce to 5% by 2050. Efficiency from 2014 onwards was then extrapolated in a linear fashion to reach 95% efficiency by 2050.

### 3.6 Capital costs of renewable technologies

Global, historical capital costs of renewable electricity generation technologies from 2015 to 2021 were extracted from IRENA's report on Renewable Power Generation Costs [15], and assumed to be representative for Viet Nam. The price for the following years were then extrapolated based on the price increase per year from 2015 to 2021. More advanced technologies such as biomass, solar PV, hydropower, and wind in the country were extrapolated until 2030, whilst less advanced technologies in the country such as CSP and geothermal were extrapolated until 2040. Values after these years were then assumed to be constant.

### 3.7 Renewable energy supply potential

The renewable energy supply potential of on-grid hydropower plants is 38 GW [16]. This was split into different sized hydropower plants based on the existing capacity of large, medium, and small hydropower plants [19]. Supply potential of off-grid hydropower is assumed to be the same as its capacity from 2015 to 2021, at 0.043 GW [5], due to lack of available data. Biomass supply potential data were collected and averaged from the Vietnam Energy Outlook [7]. From the averages, it is assumed that prices will change linearly between these data points.

## Ethics Statements

Not applicable.

## CRediT Author Statement

**Naomi Tan:** Conceptualization, Methodology, Formal analysis, Investigation, Data curation, Writing – Original draft preparation, Visualization; **John Harrison:** Supervision, Writing – Reviewing and Editing; **Mark Howells:** Supervision; **Rudolf Yeganyan:** Writing, Reviewing and Editing, Validation.

## Declaration of Competing Interest

The authors declare that they have no known competing financial interests or personal relationships that could have appeared to influence the work reported in this paper.

## Data Availability

Techno-economic dataset for long-term energy systems modelling in Viet Nam (Reference data) (Zenodo).
